# L'absence segmentaire et congénitale de la veine cave inférieure: cause rare de l'hématurie macroscopique, à propos d'un cas

**DOI:** 10.11604/pamj.2015.20.356.5978

**Published:** 2015-04-14

**Authors:** Fouad Bakloul, Mounir Lahyani, Abdelouahed Lasri, Tarik Karmouni, Khaled Elkhader, Abdelatif Koutani, Ahmed Ibnattya Andaloussi

**Affiliations:** 1 Service d'Urologie B, CHU Avicenne, Rabat, Maroc

**Keywords:** Agénésie, veine cave inférieure, hématurie, agenesis, inferior vena cava, hematuria

## Abstract

L'absence de la veine cave inférieure (VCI) est une anomalie congénitale peu fréquente, au retentissement clinique habituellement discret. Nous rapportons le cas d'un jeune homme de 19 ans, au passé de pesanteurs pelviennes chroniques, présente un épisode d'hématurie macroscopique chez qui l’échographie rénale a montré un hématome péri rénal. L'uroscanner puis l'angioscanner mettront en évidence un hématome périrénal et une agénésie segmentaire de la VCI dans sa portion sous rénale et une agénésie partielle de veine iliaque droite, avec drainage azygos et volumineux réseau de suppléance péri rénal. Nous rapportons le cas d'une agénésie du segment sous rénal de la VCI et de la veine iliaque droite.

## Introduction

L'absence de la veine cave inférieure (VCI) est une anomalie congénitale peu fréquente. L´agénésie concerne le plus souvent le segment supérieur de la VCI ou la veine dans son ensemble. L´hypertension veineuse développée dans les voies de dérivation, notamment dans la veine azygos, peut réaliser des aspects pseudo-tumoraux. Nous rapportons le cas d´une agénésie du segment sous rénal de la VCI.

## Patient et observation

**Figure 1:**
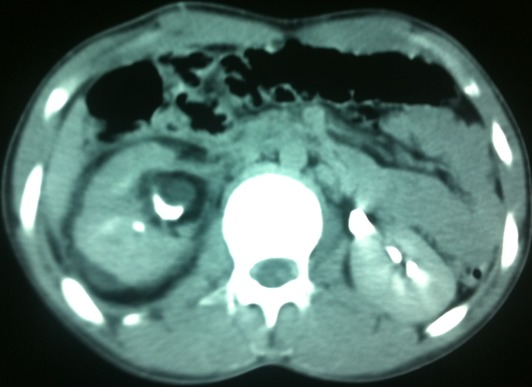
L'uroscanner montre un hématome retropéritonéal périrénal droit et un caillot pyélique droit

**Figure 2:**
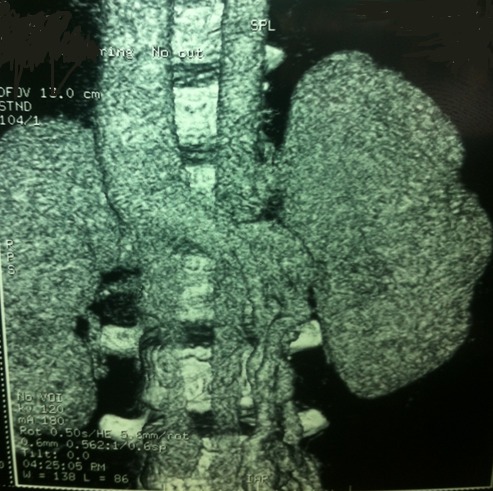
Angioscanner montrant une absence de la VCI sous-rénale

Un jeune homme de 19 ans, sans antécédents médico-chirurgicaux particuliers, présente un épisode d´hématurie macroscopique inaugural survenu à l´effort. Le patient décrit comme seul antécédent des pesanteurs pelviennes, évoluant depuis plusieurs années, aggravées par l´orthostatisme et calmées par le décubitus, qui n´ont jamais fait l´objet d´explorations. Il ne présente aucun passé uro-néphrologique, ni de maladie thromboembolique veineuse, ni autre facteur de risque cardiovasculaire. Il n´existe aucun facteur déclenchant de l´hématurie autre que l´effort. L´examen est peu contributif, montrant un sujet longiligne, en bon état général, œdème de membre inférieur droit, circulation collatérale au niveau des membres inférieurs et de la paroi abdominale. Les pouls périphériques sont perçus, sans souffle vasculaire, et l´examen du cœur et des poumons est normal l´abdomen est souple et dépressible, sans masse palpable. L´examen des organes génitaux externes retrouve une varicocèle gauche et une hydrocèle droite de faible abondance, apparemment connues du patient. Les touchers pelviens sont normaux. Biologiquement, il n´existe pas de syndrome inflammatoire (vitesse de sédimentation: 4 mm/h, protéine C réactive inférieure à 5 mg/l, fibrinogène: 2,43 g/I). Le protéinogramme ainsi que la numération formule sanguine sont normaux (leucocytes: 5600/mm3, hémoglobine: 15,10 g/dl et plaquettes: 208 000/mm3) et la fonction rénale est respectée (créatinine: 90µmol/l, urée: 4,50 mmol/l). L´analyse des urines ne retrouve pas de protéinurie. Les explorations de la coagulation et de l´hémostase sont également sans anomalie: taux de prothrombine: 82%, temps de céphaline activé du patient: 30 secondes pour un témoin à 32, protéine S: 94%, protéine C: l00%, antithrombine III: 100%, absence d´argument en faveur d´une résistance à la protéine C activée ou pour la présence d´anticoagulants circulants. L´échographie rénal puis l´uroscanner montrent un hématome retropéritonéal périrénal droit sous-capsulaire et un caillot pyélique droit en rapport avec une rupture d'une veine segmentaire intra parenchymateuse supérieure droite (Figure 1). L´écho-Doppler veineux des membres inférieurs montre une liberté du réseau veineux profond des deux membres inférieurs, sans thrombose visible ni de maladie post-phlébitique décelable. L´angio-scanner thoraco-abdomino-pelvien réalisé avec opacification veineuse par voie bipédieuse montre un système fémoral et iliaque perméable, mais une absence de la VCI sous-rénale avec un segment cave inférieur sus-rénal perméable. Le drainage veineux s´effectue préférentiellement par la veine azygos dont le calibre est augmenté à l´étage sous-diaphragmatique et au niveau thoracique, ainsi que par les veines lombaires ascendantes, les veines spermatiques et le complexe phréno-gastro-rénal (veines cardiotubérositaires postérieures et veine du pilier gauche du diaphragme). Les plexus veineux intrarachidiens ne sont pas hypertrophiés. Cet examen met également en évidence de multiples voies de dérivation à point de départ pelvien se jettent dans la veine rénale (Figure 2). L´ensemble des explorations non invasives ne retrouve incidemment aucune malformation associée tant à l´étage sous-diaphragmatique qu´au niveau thoracique. Le patient gardé sous surveillance clinique, biologique et radiologique régulière et un repos au lit stricte, l'hématurie à régresser progressivement et les urines devient claires dans les 48 heures suivants, les numérations formule sanguine n'ont pas montrés une déglobulisation, le reste du bilan biologique sans particularités. Le scanner de contrôle après une semaine du traumatisme a montré une régression de l'hématome périrénal, et le scanner après un mois montre une disparition des lésions urologiques.

## Discussion

La VCI est habituellement divisée en trois segments: un segment inférieur étendu de la naissance de la veine cave jusqu´à l´abouchement des veines rénales (segment I ou sous-rénal), un segment moyen incluant l´origine des veines rénales et le segment rétro-hépatique de la VCI jusqu´à l´abouchement des veines sus-hépatiques (segment II), et un segment supérieur comprenant l´abouchement des veines sus-hépatiques et la portion supra-hépatique de la VCI jusqu´à l´oreillette droite (segment III) [1]. L´absence de VCI est une anomalie congénitale peu fréquente, concernant le plus souvent le segment supérieur de la VCI et habituellement décelée dans l´enfance, souvent associée à d´autres malformations viscérales, principalement cardiaques (dextrocardie, malrotation) ou spléniques (asplénie ou polysplénie) [2]. Elle est ainsi retrouvée chez 0,6% des patients porteurs de malformations cardiaques [3]. Plus rarement, cette anomalie reste isolée, de découverte fortuite chez des patients asymptomatiques [4]. De rares observations ont rapporté des symptômes révélateurs à type d´hémoptysie par hypertension azygos [5, 6] ou d´insuffisance veineuse sévère des membres inférieurs avec ulcère chronique extensif [7]. Dans notre observation, l´agénésie de la veine cave inférieure se traduit cliniquement par des pesanteurs pelviennes et une hématurie macroscopique d´effort. Ces symptômes peuvent s´expliquer par la rupture d'une veine segmentaire intra-parenchymateuse secondaire à l'hyperpression veineuse. Une telle anomalie semble n´avoir jamais été rapportée au cours de l´absence de la veine cave. Des aspects pseudo-tumoraux ont déjà été décrits, mais en rapport avec une hypertension veineuse azygos majeure réalisant un aspect de masse médiastinale [8] ou de masse paravertébrale sous diaphragmatique [9]. Si, le plus souvent, l´agénésie de la VCI concerne le segment supérieur de la VCI (7), il est intéressant de noter que notre patient présente une forme rare d´absence de VCI, par agénésie du segment sous-rénal [10]. Cette constatation pourrait faire discuter une origine acquise de l´anomalie par thrombose veineuse rénale ou cave en période périnatale, comme ont pu le suggérer certains auteurs [11, 12], mais la plupart considèrent qu´il s´agit d´une anomalie du développement embryologique [13].

## Conclusion

L'hématurie macroscopique secondaire à l´absence du segment inférieur de la veine cave inférieure (VCI) est une entité pathologique rarissime voire même jamais décrite précédemment dans la littérature. Ce cas clinique nous illustre l'intérêt de penser à cette malformation devant toute hématurie et par conséquence, d'approfondir les investigations radiologiques afin de la déceler. La résolution du saignement peut survenir spontanément, ce qui fait de la surveillance une option de choix avant d'arriver à d'autres thérapeutiques invasives.
